# Antibody dependent enhancement of frog virus 3 infection

**DOI:** 10.1186/1743-422X-7-41

**Published:** 2010-02-18

**Authors:** Heather E Eaton, Emily Penny, Craig R Brunetti

**Affiliations:** 1 Department of Biology, Trent University, Peterborough, ON, K9J 7B8, Canada

## Abstract

**Background:**

Viruses included in the family *Iridoviridae *are large, icosahedral, dsDNA viruses that are subdivided into 5 genera. Frog virus 3 (FV3) is the type species of the genus *Ranavirus *and the best studied iridovirus at the molecular level. Typically, antibodies directed against a virus act to neutralize the virus and limit infection. Antibody dependent enhancement occurs when viral antibodies enhance infectivity of the virus rather than neutralize it.

**Results:**

Here we show that anti-FV3 serum present at the time of FV3 infection enhances infectivity of the virus in two non-immune teleost cell lines. We found that antibody dependent enhancement of FV3 was dependent on the Fc portion of anti-FV3 antibodies but not related to complement. Furthermore, the presence of anti-FV3 serum during an FV3 infection in a non-immune mammalian cell line resulted in neutralization of the virus. Our results suggest that a cell surface receptor specific to teleost cell lines is responsible for the enhancement.

**Conclusions:**

This report represents the first evidence of antibody dependent enhancement in iridoviruses. The data suggests that anti-FV3 serum can either neutralize or enhance viral infection and that enhancement is related to a novel antibody dependent enhancement pathway found in teleosts that is Fc dependent.

## Background

Following a viral infection an immune response is elicited by the host, which includes both an innate and adaptive response. During the adaptive immune response, antibodies are produced that are designed to recognize and neutralize a pathogen. Typically, viral antibodies neutralize a virus by preventing the attachment of specific cell surface receptors with viral glycoproteins, while also activating the complement system. However, not all antibodies serve to reduce infectivity. Antibody dependent enhancement (ADE) occurs when viral antibodies enhance infectivity of a virus by promoting the attachment of viral particles to cells. Virus specific antibodies bind to viral particles to form complexes that can bypass normal routes of viral attachment and entry. The virus+antibody complex allows for increased viral entry or infection of cells that would not normally become infected. Virus+antibody complexes therefore result in a more efficient infection than with virus alone.

There are several mechanisms of how ADE can occur. The most common mechanism of ADE is Fc receptor (FcR)-dependent [1]. In FcR-dependent ADE the virus+antibody complex binds to cells containing FcRs on their surface. The interaction is mediated between the exposed Fc region of the antibody (from the virus+antibody complex) and the FcR on the cell surface. FcRs are found on a wide variety of cells of the immune system, including macrophages, B cells, neutrophils, monocytes, and granulocytes [2,3]. However, since not all cells that exhibit ADE are immune cells, another mechanism must be responsible for ADE in non-FcR bearing cells. Complement-mediated ADE is not exclusive to FcR bearing cells because complement receptors are found on a large variety of cell types [4]. Complement-mediated ADE occurs via binding between the Fc region of antibodies and C1q [1]. This can result in a variety of outcomes including the activation of complement, which causes complement C3 fragment and viral surface proteins to bind and promote viral attachment. C1q can also enhance virus attachment by binding to C1qR on the cell surface, which brings the virus into close proximity to cells.

ADE can result in increased viral pathogenesis because it enhances a virus's ability to bind to cells. It therefore can result in increased severity of disease. This was first shown with dengue virus where a second infection resulted in an increased number of infected cells and higher levels of virus production [5,6]. An *in vitro *study suggested that the mechanism behind ADE in dengue virus was FcR-dependent [7-9]. Dengue virus titer was enhanced dramatically through the binding of the virus+antibody complex to FcRs found on cells of the immune system [7-9].

While ADE has been demonstrated for many RNA viruses, only a few DNA virus families, including poxviruses [10] and herpesviruses [11-13] have been shown to use ADE as a mechanism of infection. While it is suggested that they most likely use FcR-dependent ADE [1], little is actually known about the mechanism of ADE in the large DNA viruses. We decided to determine if viruses from the family *Iridoviridae *use ADE as a mechanism of infection. Viruses in the family *Iridoviridae *are large (~120-200 nm), icosahedral viruses that contain a linear, double-stranded DNA genome. Iridovirus infections appear to be restricted to invertebrates (*Iridovirus*, *Chloriridovirus*) and poikilothermic vertebrates (*Lymphocystivirus*, *Ranavirus*, *Megalocytivirus*) [14]. Although iridoviruses are large DNA viruses, very little is known about their biology. Using frog virus 3 (FV3; *Ranavirus*) as a model virus, we propose to investigate whether ADE occurs in viruses of the family *Iridoviridae*, specifically in the *Ranavirus *genus.

## Results

### ADE increases FV3 infection in teleost cells

**Figure 1:**
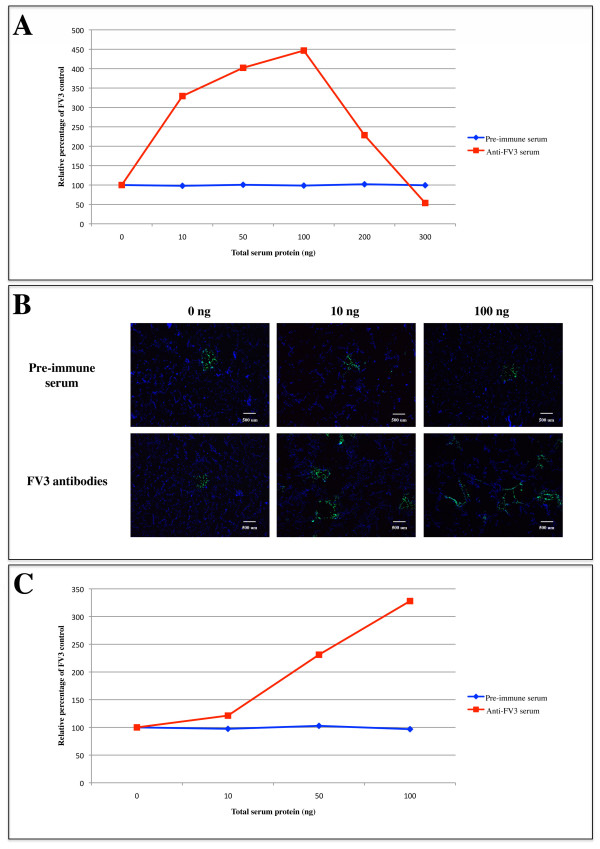
**ADE occurs during an FV3 infection in teleost cells**. FV3 (~50 PFU) was incubated alone, with rabbit anti-FV3 serum, or rabbit pre-immune serum for 1 hour at 4°C and was then added to BF-2 or FHM cells. (A) Two hours post-infection, BF-2 cells were overlaid and 48 hours later the plaques were stained with crystal violet and were counted. Plaque numbers are shown as a relative percentage of a control FV3 infection in the absence of serum. (B) Forty-eight hours post-overlay BF-2 cells were stained by indirect immunofluorescence using anti-FV3 serum (green) and DAPI (nuclei - blue). (C) FV3+anti-FV3 serum or FV3+rabbit pre-immune serum complexes were added to FHM cells and 24 hours later plaques were visualized by indirect immunofluorescence and counted. Plaque numbers were expressed as a relative percentage compared to FHM cells infected with FV3 only. All experiments were completed in 3 independent trials and mean plaque numbers are shown.

In order to investigate whether ADE occurs during an FV3 infection, FV3 was pre-incubated with either rabbit anti-FV3 serum (FV3+anti-FV3 serum) or rabbit pre-immune serum (FV3+pre-immune serum). The FV3+anti-FV3 serum and FV3+pre-immune serum complexes were added to BF-2 (teleost fibroblast) or FHM (teleost epithelial) cells. Two hours post-infection, BF-2 and FHM cells were overlaid with agarose and 48 hours later the number of plaques produced by the virus were counted and compared to the number of plaques from an FV3 only control infection. All experiments were repeated in at least 3 independent trials and mean results are shown (Figure 1A, Additional file 1A). The addition of 100 ng of anti-FV3 serum to the virus in BF-2 cells resulted in an ~300% increase in the number of plaques compared to pre-immune serum (Figure 1A). Following the addition of the highest concentration of anti-FV3 serum (300 ng), we found that the plaque number was reduced as compared to an FV3 control indicating that at high concentrations, anti-FV3 serum can neutralize the infection (Figure 1A). Infection of cells by FV3+anti-FV3 serum complexes also increased the number of plaques in BF-2 cells compared to an infection with FV3+pre-immune serum complexes as seen by immunofluorescence (Figure 1B). Anti-FV3 serum staining revealed small plaques in cells infected with FV3+ pre-immune serum complexes while cells infected with FV3+anti-FV3 serum complexes showed more frequent and larger sized plaques (Figure 1B). A control experiment in which pre-immune serum or anti-FV3 serum were added to cells without FV3 resulted in an absence of plaques. In another teleost cell line (FHM), we observed a greater than 200% increase in the number of plaques in the presence of 100 ng of anti-FV3 serum (Figure 1C, Additional file 1B). This data suggests that ADE occurs during an FV3 infection in teleost cells.

### Anti-FV3 serum neutralizes infection in BGMK cells

**Figure 2:**
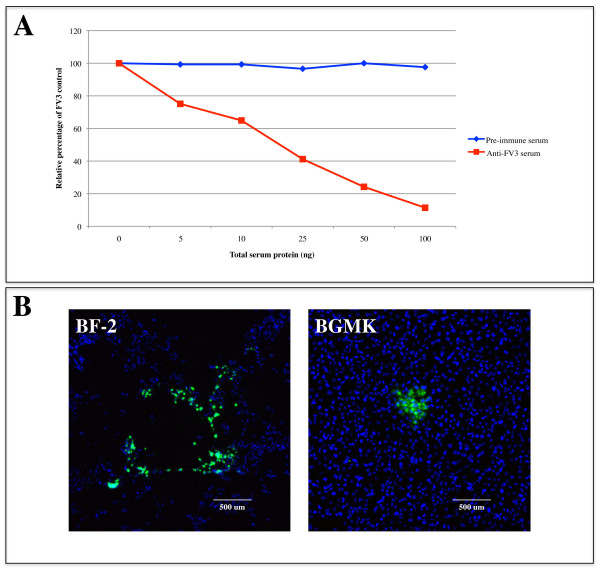
**Rabbit anti-FV3 serum neutralizes an FV3 infection in a mammalian cell line**. Rabbit anti-FV3 serum (0-100 ng) or rabbit pre-immune serum (0-100 ng) was incubated with FV3 (~50 PFU) before being added to BGMK or BF-2 cells for 2 hours. Once infected, BGMK cells were incubated at 28°C with 5% CO_2_. The cells were subsequently overlaid with agarose. (A) Forty-eight hours post-overlay BGMK cells underwent indirect immunofluorescence and plaques were counted. Plaque numbers from 3 independent trials were counted and mean plaque values were compared to BGMK cells infected with FV3 only and values are shown as a relative percentage. (B) Forty-eight hours post-overlay, BGMK and BF-2 cells were processed for indirect immunofluorescence using rabbit anti-FV3 serum (green) and DAPI (nuclei - blue).

FV3 replicates in a variety of cell types including cells of mammalian origin [15]. In order to determine whether the ADE phenomenon occurs in mammalian cells along with teleost cells, we pre-incubated FV3 with anti-FV3 serum or pre-immune serum and FV3+anti-FV3 serum or FV3+pre-immune serum complexes were added to BGMK (mammalian fibroblast) and BF-2 (teleost fibroblast) cells and the cells were overlaid with agarose. Forty-eight hours later, the overlay was removed and indirect immunofluorescence was used to visualize plaques. In contrast to teleost cells (Fig 1A), the addition of 100 ng of anti-FV3 serum to mammalian cells resulted in an ~90% reduction in the number of plaques compared to the pre-immune serum control (Figure 2A, Additional file 2). Furthermore, the plaques produced by an FV3 infection in BGMK cells were considerably smaller than those seen in BF-2 cells (Figure 2B). These results suggest that in mammalian cells anti-FV3 serum does not enhance an FV3 infection but instead neutralizes it. Thus, ADE does not occur in an FV3 infection in mammalian fibroblast cells but does occur in teleost fibroblast cells.

### Addition of pre-immune serum to BF-2 cells inhibits ADE of FV3 infectivity

**Figure 3:**
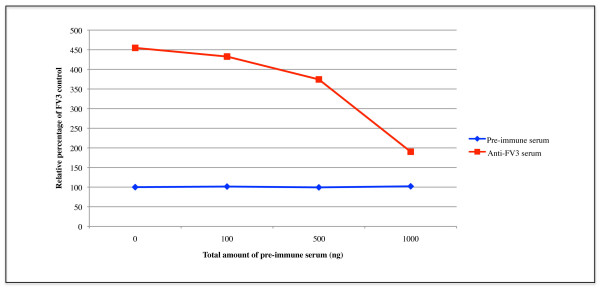
**Addition of rabbit pre-immune serum to BF-2 cells inhibits ADE**. FV3 (~50 PFU) was incubated with either 50 ng of rabbit anti-FV3 serum or 50 ng rabbit pre-immune serum. FV3+anti-FV3 serum or FV3+pre-immune serum complexes were added to BF-2 cells along with varying amounts of pre-immune serum (0-1000 ng). Cells were overlaid and plaques were visualized with crystal violet. Mean plaques values from 3 independent trials were obtained and mean values were expressed as a relative percentage to BF-2 cells infected with FV3 in the absence of anti-FV3 serum or pre-immune serum.

In order to determine if the ADE was specific to anti-FV3 serum, we challenged cells with non-specific rabbit serum to act as a competitive inhibitor of anti-FV3 serum. FV3+anti-FV3 serum (50 ng) or FV3+pre-immune serum (50 ng) complexes were allowed to form and were then added to cells along with increasing amounts (0-1000 ng total serum) of non-specific rabbit serum. Infected cells were incubated for 2 hours and overlaid with agarose. Forty-eight hours later the plaques were counted. The addition of increasing amounts of non-specific competitive serum resulted in a reduction in ADE (Figure 3, Additional file 3). At the highest concentration of competitor, 1000 ng (20-fold excess), there was an almost 300% reduction of FV3 ADE (Figure 3) as compared to cells where pre-immune serum was not added (Figure 3: 0 ng). These results suggest that the non-specific serum acts as a competitive inhibitor presumably binding to cell surface components that mediate ADE.

### Protein A eliminates ADE of FV3 infectivity

**Figure 4:**
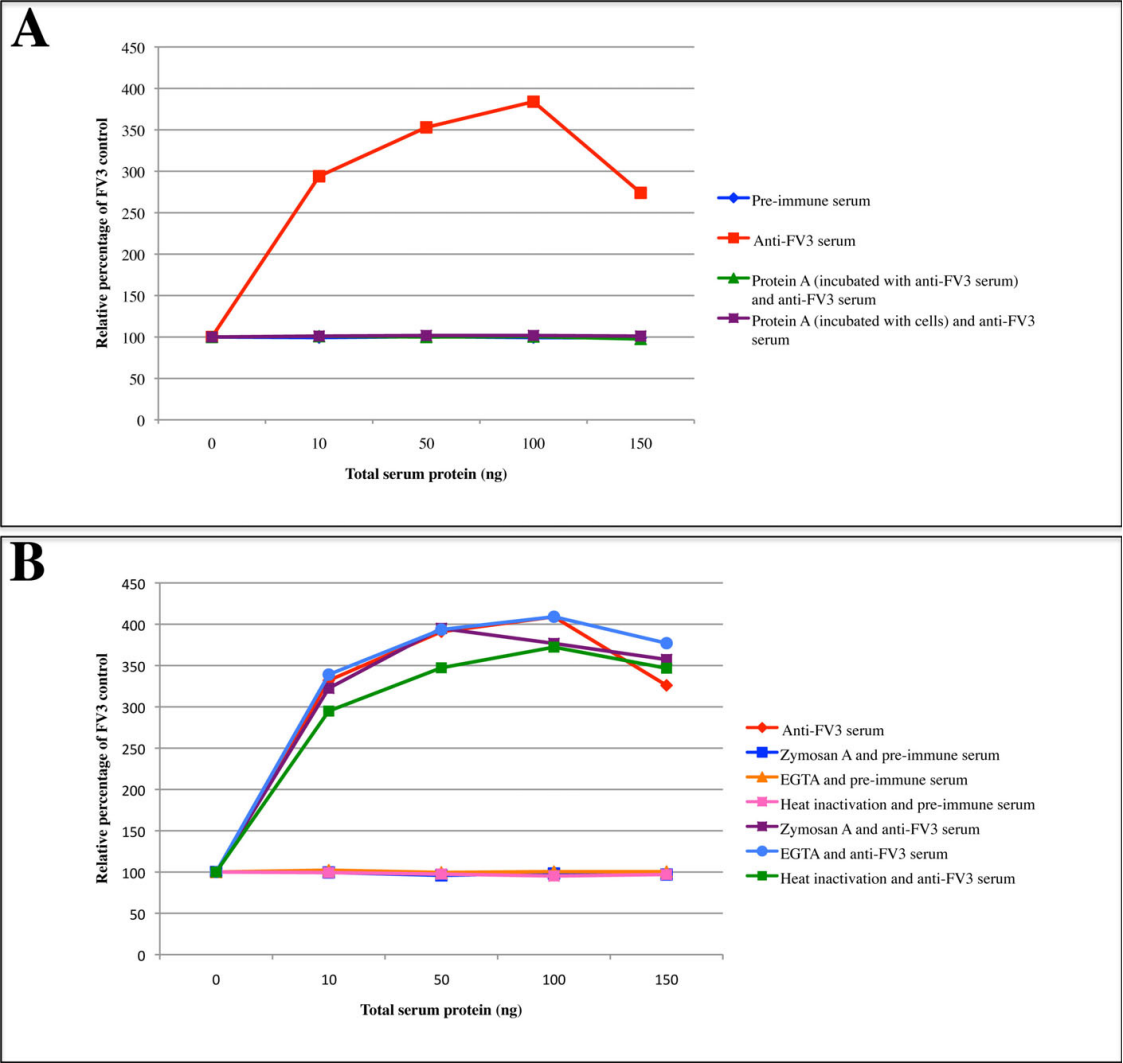
**ADE in FV3 is Fc-dependent and independent of complement**. (A) Protein A (300 μg/mL) was either incubated with BF-2 cells or anti-FV3 serum and pre-immune serum for 30 minutes at room temperature. FV3 (~50 PFU) was incubated with anti-FV3 serum or pre-immune serum and was then added to BF-2 cells (+/- protein A) and were overlaid 2 hours later. FV3 (~50 PFU) was incubated with anti-FV3 serum or pre-immune serum (+/- protein A) and then was added to BF-2 cells and were overlaid 2 hours later. Forty-eight hours post-overlay plaques were counted and expressed as a relative percentage of a control FV3 infection. (B) Rabbit anti-FV3 serum or rabbit pre-immune serum were heat-inactivated or incubated with zymosan A or EGTA for one hour before the addition of FV3 (~50 PFU). FV3+anti-FV3 serum or FV3+pre-immune serum complexes were added to BF-2 cells, which were subsequently overlaid. Forty-eight hours post-overlay, plaques were counted and were compared as a relative percentage to BF-2 cells infected with FV3 in the absence of serum. Experiments were completed in at least 3 independent trials with mean plaque values shown.

Since ADE occurs in an FV3 infection in teleost cells, we next wanted to determine if the Fc portion of anti-FV3 antibodies mediates ADE. Protein A binds to the Fc region of an antibody, thereby blocking binding between the Fc region of the antibody and FcRs and complement proteins on the cell surface. Protein A (300 μg/mL) was pre-incubated with rabbit anti-FV3 serum or rabbit pre-immune serum followed by the addition of FV3. FV3+anti-FV3 serum/(+/-)protein A or FV3+pre-immune serum/(+/-)protein A complexes were added to cells and plaques were counted 48 hours later. The addition of protein A to anti-FV3 serum completely abolished the ADE in BF-2 cells (Figure 4A, Additional file 4A). Note that the abolishment in infectivity by protein A is so complete that the virus samples incubated with protein A are indistinguishable from the pre-immune control (Figure 4A). These results suggest that the Fc portion of anti-FV3 antibodies is responsible for mediating ADE. However, the addition of protein A to serum can sometimes result in aggregation of the antibodies, thereby reducing the amount of available antibody. In order to avoid this issue we incubated 300 μg/mL of protein A on BF-2 cells for 30 minutes prior to the addition of FV3+anti-FV3 serum or FV3+pre-immune serum complexes. Forty-eight hours post-infection plaques were counted. The addition of protein A to BF-2 cells prior to addition of anti-FV3 serum allowed FV3+anti-FV3 serum complexes to form. However, we obtained a complete abolishment of enhancement similar to that seen when protein A was pre-incubated with FV3+anti-FV3 serum (Figure 4A, Additional file 4B). These results suggest that ADE of FV3 infectivity can be inhibited by protein A and is likely Fc-dependent.

### ADE of FV3 infectivity is complement-independent

To determine whether FV3 ADE was complement-dependent, anti-FV3 serum and pre-immune serum were heat-inactivated to inactivate complement, or incubated with either EGTA or zymosan A. EGTA is a chelator that inhibits the classical complement pathway, while zymosan A disrupts the alternative complement pathway. Treated anti-FV3 serum or pre-immune serum was incubated with FV3 before addition to BF-2 cells. Forty-eight hours post-overlay plaques were counted. Infection by the FV3+anti-FV3 serum complexes treated with heat-inactivation, EGTA, or zymosan A did not reduce the ADE of FV3 infectivity as compared to the untreated FV3+anti-FV3 serum control (Figure 4B, Additional file 5A, 5B, 5C). Regardless of whether high or low levels of complement activity were present at the time of infection, enhancement was not affected by complement inhibitors suggesting that inactivation of complement does not disrupt ADE of FV3 infectivity.

### Fc binding proteins on teleost cells

**Figure 5:**
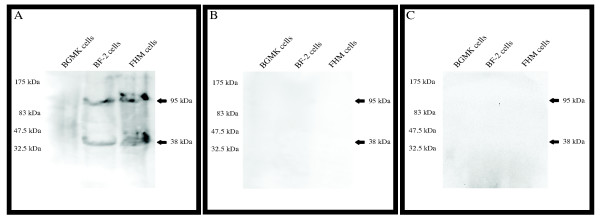
**Rabbit serum binds to two proteins (38 kDa and 95 kDa) on teleost cells**. BGMK, BF-2, and FHM cells were harvested and run on a 10% SDS acrylamide gel. Proteins were transferred from the gel to a PVDF membrane. The membrane was probed with (A) rabbit pre-immune serum, (B) rabbit pre-immune serum pre-incubated with protein A, or (C) no primary serum was added. Proteins were then visualized using peroxidase conjugated goat anti-rabbit IgG and chemiluminescence.

The ability of anti-FV3 serum to neutralize an infection in BGMK cells (mammalian fibroblast) and enhance infection in BF-2 and FHM cells (teleost fibroblast and epithelial respectively) suggests that teleost cells may contain an Fc-binding component absent from BGMK cells. Since the pre-immune control serum was able to act as a competitive inhibitor (Figure 3), it suggests that there must be a specific component on teleost cells that the serum is binding to. A western blot containing BGMK, BF-2, and FHM cellular extracts was probed with rabbit pre-immune serum to determine if the serum bound to any cellular proteins. While rabbit serum was unable to bind to any proteins in BGMK cells (which do not undergo ADE), two bands at 38 and 95 kDa were detected in BF-2 and FHM cells probed with rabbit pre-immune serum (Figure 5A). No bands were detected either on a control blot where the primary rabbit serum was omitted (Figure 5C) or on a membrane where pre-immune serum was pre-incubated with protein A (Figure 5B), which binds to the Fc portion of the antibody. These data suggest that the Fc portion of the antibody bound to the 38 kDa and 95 kDa proteins and that the variable region of the antibody does not mediate this interaction. The experiment was repeated several times using an unrelated rabbit serum and results consistent with Figure 5 were obtained (data not shown). This data suggests that an Fc binding component specific to fibroblast and epithelial teleost cells may play a role in ADE of an FV3 infection.

## Discussion

While ADE has been previously reported for some large DNA viruses, including herpesviruses [11-13] and poxviruses [10], no studies to date have demonstrated ADE as a mechanism to enhance infections in iridoviruses. This paper provides the first evidence of ADE in iridoviruses, specifically in the *Ranavirus *genus.

In this study, anti-FV3 serum demonstrated the ability to either neutralize or enhance an FV3 infection depending on the cell line. Although the infection was less efficient in mammalian cells, FV3 exhibited the ability to enter the cell and spread as was revealed by the presence of numerous plaques post-infection. The addition of anti-FV3 serum to an FV3 infection in BGMK cells dramatically reduced plaque number and size demonstrating the ability of the anti-FV3 serum to neutralize the infection in a mammalian cell line. However, the opposite effect was seen in teleost (BF-2 and FHM) cell lines. ADE often occurs with neutralizing antibodies at sub-neutralizing concentrations and differences in the interaction between virus and antibody can lead to either neutralization or enhancement of a viral infection [16]. Furthermore, enhancement of an infection is particularly sensitive to this interaction and can also involve the target cell. Our data suggests that the anti-FV3 serum used in this study possess both neutralizing and enhancing activity, depending on various factors including the cell type and the concentration of antibody.

Ranaviruses, including FV3, have been isolated from a variety of species including fish and amphibians. While FV3 has never been isolated from fish *in vivo*, other closely related (over 98% sequence identity of the major capsid protein [17]) ranaviruses, including epizootic haematopoietic necrosis virus (EHNV) and Bohle virus (BIV) infect fish and infection can result in high levels of morbidity and mortality [18-20]. FV3 shows high levels of infectivity in fish cell lines *in vitro *[21-23]; therefore two fish cells lines (BF-2 and FHM) were used during this study. While there are many differences from mammalian immune systems, the immune systems of fish and amphibians are fundamentally similar to mammals with both innate immunity and adaptive immune functions [24,25]. However, immunoglobins of lower vertebrates are currently poorly understood as compared to those of mammals. Fish were the first group to have demonstrated antibody activity and have one predominant Ig isotype, an IgM-like tetrameric molecule [26]. Amphibians have several isotypes including IgY, which is the predominant isotype in amphibians and is considered the functional equivalent to mammalian IgG [27-29]. However, the adaptive immune system of mammals is fundamentally similar to that of lower vertebrates and is characterized by T cell receptors, Ig, and the major histocompatibility complex (MHC). Lower vertebrates also rely heavily on non-specific defense systems for pathogen defense and therefore the innate immune system of lower vertebrates, including complement, is diverse and similar to that of higher vertebrates. We therefore suspect that the mechanisms behind ADE in fish and amphibians will be similar to that of mammals. While we feel the mechanisms behind ADE to be similar between fish, amphibians, and mammals, there are some inherent differences between the immune systems of each species. It will be important to confirm these experiments in the future using sera from either immunized frogs or fish.

Common mechanisms behind ADE can be dependent on either complement or FcRs. Our results suggest that complement pathways (classical or alternative) do not play a role in the enhancement of FV3 infection by anti-FV3 serum. However, protein A eliminated any enhancement of the anti-FV3 serum suggesting the mechanism behind FV3 ADE to be FcR-dependent. Many virus families, including other DNA viruses, enhance viral infection through the binding of the Fc region of anti-viral antibodies to FcR on the surface of cells of the immune system [30-37]. However, this result is intriguing because both cell lines that exhibited ADE (BF-2 and FHM) in this study are non-immune (fibroblast and epithelial, respectively) cell lines that should lack FcR on the cell surface [2,3,9]. While recent research suggests that teleosts and amphibians possess both FcR homologs and novel immune-type receptors (NITRs) [38-42], little is known about their tissue distribution and role in innate immunity. FcRs in humans are a variety of sizes that can range from 40 kDa to over 70 kDa [43-46], while one previously identified FcR in fish was predicted to be ~33 kDa in size [40]. We identified two proteins (38 kDa and 95 kDa) in teleost cells (but not in a mammalian cell line) that bound to the Fc region of rabbit antibodies. The molecular weight of these proteins does not rule out the possibility that they may function as novel FcRs in teleosts. While we do not specifically know what the anti-FV3 serum is binding to mediate ADE, the results suggest that proteins specific to teleost cells bind to the Fc region of antibodies potentially mediating ADE.

Iridovirus infections of increased pathogenicity have been recently observed in several wild and cultivated fish and amphibian species [17,47,48]. Specifically, ranavirus infections pose a potential threat to amphibians and have been implicated in the widespread decline of worldwide amphibian populations [48,49]. There have recently been increasing reports of ranavirus infections, with both the severity of infections and the number of species infected increasing [17,50-55]. While evidence suggests that an iridovirus infection mounts a strong immune response [56,57], this does not eliminate the possibility that viral infection can be enhanced under certain circumstances. Although humoral immunity is required for protection against viruses, antibodies at sub-neutralizing concentrations may enhance, rather than protect against infection [16]. It is also possible that the virus utilizes ADE as a method for more efficient entry. Regardless of whether a strong immune response is mounted, ADE may promote increased entry or entry into cells not usually infected. In particular, ADE in immunocompromised individuals may allow for increased infection. Furthermore, the link between ADE *in vitro *and *in vivo *currently remains elusive. For instance, ADE of dengue viruses has been well documented and extensively characterized *in vitro*, but *in vivo *studies remain unclear and controversial [58-60]. It will be important for future experiments to confirm these *in vitro *studies using live fish and frogs. Ranavirus infections are spreading rapidly worldwide, however, the reasons behind this rapid spread are currently unknown and are most likely complex. While FV3 ADE has yet to be demonstrated *in vivo*, a strong second infection of FV3 may explain the increased severity and prevalence of ranavirus infections. Therefore, ADE may represent a potential hypothesis for the recent emergence and increased severity of ranavirus infections.

## Conclusions

This study demonstrates for the first time that FV3, an iridovirus, utilizes ADE to increase infection *in vitro*. The anti-FV3 serum used in this study both enhanced and neutralized a viral infection depending on cell type and concentration. The mechanism behind enhancement was found to be independent of complement but dependent upon the Fc region of anti-FV3 antibodies. The addition of protein A to either anti-FV3 serum or teleost cells completely abolished ADE. This result was surprisingly because two non-immune cell lines most likely lacking FcR were used during this experiment. Our results suggests that the Fc region of anti-FV3 antibodies may promote viral entry through novel Fc binding activity on teleost cells.

## Methods

### Cell lines and virus

Bluegill fry (BF-2) cells were obtained from American Type Culture Collection (ATCC, Manassas, VA) and were grown at 28°C in 5% CO_2 _in Eagle's Minimal Essential medium with Earle's balanced salts (EMEM; HyClone, Ottawa, ON) and 2 mM L-glutamine supplemented with 10% fetal bovine serum (FBS), 1.0 mM sodium pyruvate, 0.1 mM nonessential amino acids, and antibiotics (100 U/mL penicillin and 100 g/mL streptomycin). Baby green monkey kidney (BGMK) cells were obtained from ATCC and were maintained in Dulbecco's modified Eagle's medium (DMEM; HyClone) supplemented with 7% FBS, 2 mM L-glutamine, penicillin (100 U/mL), and streptomycin (100 g/mL) at 37°C with 5% CO_2_. We have previously characterized an FV3 infection in BGMK cells [61]. Fathead minnow (FHM) cells were also obtained from ATCC and were maintained at 30°C in minimum essential medium with Hanks' salts (MEM; Invitrogen, Burlington, ON) supplemented with 10% FBS, penicillin (100 U/mL), and streptomycin (100 g/mL). FV3 was obtained from ATCC and rabbit anti-FV3 serum and rabbit pre-immune serum were kindly provided by V.G. Chinchar (University of Mississippi Medical Center, Jackson, MS). Once BGMK cells were infected with FV3 they were incubated at 28°C with 5% CO_2_.

### ADE plaque assay

FV3 (~50 PFU) was mixed with either rabbit anti-FV3 serum or control rabbit pre-immune serum (0 ng, 10 ng, 50 ng, 100 ng, 200 ng, and 300 ng total serum protein) for a final volume of 100 μL in media and was incubated for 1 hour at 4°C. The FV3+anti-FV3 serum or FV3+pre-serum complexes were then added to BF-2, FHM, or BGMK cells grown to 90% confluence in 6-well plates. BF-2 and BGMK cells were overlaid with 2% agarose 2 hours post-infection. Forty-eight hours post-overlay cells were either stained with crystal violet (0.05%) or underwent indirect immunofluorescence and plaques were counted. FHM cells were incubated for 24 hours and indirect immunofluorescence was carried out and plaques were counted.

### Pre-immune serum challenge

Rabbit anti-FV3 serum (50 ng total serum protein) or rabbit pre-immune serum (50 ng total serum protein) were mixed with FV3 (~50 PFU) in a final volume of 100 μL in EMEM and were incubated for 1 hour at 4°C. Pre-immune serum was added to BF-2 cells grown to 90% confluence in 6-well dishes for final concentrations of 0 ng/μL, 0.1 ng/μL, 0.5 ng/μL, and 1 ng/μL. FV3+anti-FV3 serum or FV3+pre-immune serum complexes were added to BF-2 cells containing pre-immune serum and were incubated for 2 hours. Cells were overlaid with 2% agarose and 48 hours post-overlay crystal violet (0.05%) was added to cells and plaques were counted.

### Inhibition of Fc and complement

Rabbit anti-FV3 serum (0-150 ng total serum protein) or rabbit pre-immune serum (0-150 ng total serum protein) were incubated with protein A (300 μg/mL; Sigma, Oakville, ON) or EGTA (0.05 M) for 30 minutes at room temperature, zymosan A (20 mg/mL; Sigma) for 1 hour at 37°C, or were heat-inactivated at 56°C for 30 minutes. Approximately 50 PFU of FV3 was added and the FV3+anti-FV3 serum or FV3+pre-immune serum complexes were brought up to a final volume of 100 μL with serum-free EMEM. An ADE plaque assay in BF-2 cells was then performed. Protein A (300 μg/mL) was incubated with BF-2 cells for 30 minutes at room temperature. Cells were washed several times with PBS and 50 PFU of FV3 previously incubated with 0-150 ng anti-FV3 serum or pre-immune serum for one hour at 4°C were added to the cells. An ADE plaque assay using 0 ng, 10 ng, 50 ng, 100 ng, and 150 ng of rabbit anti-FV3 serum or control rabbit pre-immune serum was then performed.

### Indirect immunofluorescence

Cells were fixed for 10 minutes in 3.7% paraformaldehyde in phosphate buffer saline (PBS). Following several washes, cells were incubated in block buffer (5% bovine serum albumin (BSA) (w/v), 50 mM Tris HCl (pH 7.4), 150 mM NaCl, 0.5% NP-40 (v/v)) overnight at 4°C. Cells were incubated with rabbit anti-FV3 serum (dilution: 1/1000) for one hour at room temperature. Cells were then incubated in FITC-conjugated goat anti-rabbit immunoglobulin G (IgG) (dilution: 1/100) (Jackson ImmunoResearch Inc., West Grove, PA) and Texas Red^®^-X Phalloidin (dilution 1/40) (Invitrogen) for one hour at room temperature. Finally, cells were incubated for 2 minutes in the nucleic acid stain DAPI (Invitrogen) diluted to 300 nM in PBS. Immunofluorescence was detected using a Leica DM6000 B fluorescent microscope (Leica, Wetzlar, Germany). Images were assembled using Adobe Photoshop CS4 (Adobe, San Jose, CA).

### Western Blotting

BGMK, BF-2, and FHM cells grown to 100% confluence in a 6-well dish were scraped into the media and centrifuged at 10,000 × g for 5 minutes. The supernatant was removed and the cells were re-suspended in Laemmli reducing buffer [62]. Cell lysates were boiled and proteins were separated on a 10% polyacrylamide gel using sodium dodecyl sulfate (SDS) running buffer (125 mM Tris, 1.25 M glycine, 0.5% SDS). Following electrophoresis, the proteins were transferred from the gel to a polyvinylidene difluoride (PVDF) membrane using a semi-dry transfer apparatus (FisherBiotech, Pittsburgh, PA). The membrane was blocked overnight at 4°C in TBST buffer (140 mM NaCl, 24 mM Tris (pH 7.4), 0.2% Tween^® ^20, 3 mM KCl) containing 5% non-fat milk powder. The membrane was incubated without primary serum, rabbit pre-immune serum (dilution 1:1000), rabbit pre-immune serum pre-incubated with 300 μg/mL protein A for 30 minutes at room temperature (dilution 1:1000), or a second unrelated rabbit pre-immune serum (dilution 1:1000) for 1 hour shaking at room temperature. The membrane was washed several times then incubated for 1 hour shaking at room temperature in peroxidase-conjugated AffiniPure F(ab')2 fragment goat anti-rabbit IgG (Jackson ImmunoResearchInc.) diluted 1/10,000. The membrane was washed several times and proteins were detected by applying Chemiluminescence Reagent Plus (Perkin-Elmer, Boston, MA) to the membrane as per the manufacture's protocol. The images were then viewed using a Genius^2 ^Bio Imaging System (Syngene, Frederick, MD).

## Competing interests

The authors declare that they have no competing interests.

## Authors' contributions

HEE performed the research and helped to draft the manuscript. EP helped to perform the research. CRB conceived the study and participated in its design and coordination and helped draft the manuscript. All authors read and approved the final manuscript.

## Supplementary Material

Additional file 1 **ADE occurs during an FV3 infection**. ADE occurs during an FV3 infection in (A) BF-2 and (B) FHM cells. Original plaque numbers and standard error from three individual experiments are shown. Click here for file

Additional file 2 **Rabbit anti-FV3 serum neutralizes an FV3 infection in BGMK cells**. Original plaque numbers and standard error from three individual experiments are shown. Click here for file

Additional file 3 **Addition of rabbit pre-immune serum to BF-2 cells inhibits ADE**. Original plaque numbers and standard error from three individual experiments are shown. Click here for file

Additional file 4 **ADE in FV3 is Fc-dependent**. Protein A incubated with either (A) anti-FV3 serum or (B) BF-2 cells abolished ADE in BF-2 cells. Original plaque numbers and standard error from three individual experiments are shown. Click here for file

Additional file 5 **ADE in FV3 is complement independent**. Treatment of anti-FV3 serum with (A) heat inactivation, (B) EGTA, and (C) zymosan A did not affect ADE. Original plaque numbers and standard error from three individual experiments are shown. Click here for file
